# Historical Epidemiology of the Second Cholera Pandemic: Relevance to Present Day Disease Dynamics

**DOI:** 10.1371/journal.pone.0072498

**Published:** 2013-08-22

**Authors:** Christina H. Chan, Ashleigh R. Tuite, David N. Fisman

**Affiliations:** Division of Epidemiology, Dalla Lana School of Public Health, University of Toronto, Toronto, Ontario, Canada; Université Catholique de Louvain, Belgium

## Abstract

Despite nearly two centuries of study, the fundamental transmission dynamic properties of cholera remain incompletely characterized. We used historical time-series data on the spread of cholera in twelve European and North American cities during the second cholera pandemic, as reported in Amariah Brigham’s 1832 *A Treatise on Epidemic Cholera*, to parameterize simple mathematical models of cholera transmission. Richards growth models were used to derive estimates of the basic reproductive number (R_0_) (median: 16.0, range: 1.9 to 550.9) and the proportion of unrecognized cases (mean: 96.3%, SD: 0.04%). Heterogeneity in model-generated R_0_ estimates was consistent with variability in cholera dynamics described by contemporary investigators and may represent differences in the nature of cholera spread. While subject to limitations associated with measurement and the absence of microbiological diagnosis, historical epidemic data are a potentially rich source for understanding pathogen dynamics in the absence of control measures, particularly when used in conjunction with simple and readily interpretable mathematical models.

## Introduction

Cholera is an infectious diarrhoeal disease caused by *Vibrio cholerae*. The earliest western record of cholera dates back to at least 16^th^ century, when cases were observed in India [1], but global pandemics of “asiatic cholera” were first documented in 1817. Cholera can be endemic or epidemic, and is a disease with pandemic potential, with pandemics occurring as a result of genetic reassortment microbial strains [2]; the seventh recorded cholera pandemic occurred in the 1960s and cholera remains endemic in many countries [3]. Cholera is treatable with oral rehydration therapy and preventable with adequate sanitation and water treatment, and cholera epidemics have not been seen in high income countries since the early 20^th^ century [3]; however, the disease remains a major global threat, with an estimated 3–5 million cases and 100000–120000 deaths annually [4]. In recent years, Africa has accounted for over 90% of cases reported to the World Health Organization (WHO) globally, with majority of the remaining cases reported from low and middle income countries in Asia and South America [5], [6].

Cholera swept across Europe for the first time in 1831 during the second cholera pandemic (the first (1817) pandemic reached only as far west as the Caspian Sea). The pandemic originated in India in 1826 and moved along trade and military campaign routes to Central Asia, the Middle East, Europe from east to west across the Baltic states, and eventually to North America [1]. Notwithstanding this geographic march, cholera was not widely believed to be contagious prior to John Snow’s work on the 1854 London cholera outbreak. Prevalent models for disease spread, some of which lasted until the end of the 19^th^ century, included the diffusion of a poisonous “miasma”, spread by tiny, invisible insects, and hypotheses related to the disruption of the earth’s magnetic field or atmosphere [7]. While the dispatch of expert commissions to study the progress of the disease in far-off locales [8] and the deployment of military cordons to prevent the disease’s spread in eastern and central Europe [1] suggest an implicit recognition of transmissibility, at least by civil authorities, medical professionals tended to deride those who suggested cholera might be contagious as superstitious and unsophisticated. As such, limited attempts were made to control the spread of disease using interventions that could be considered truly effective, and 19^th^ century epidemics may, consequently, provide a snapshot of the true “natural history” of cholera.

Notwithstanding nearly two centuries of study by epidemiologists, the fundamental dynamic properties of cholera remain poorly characterized. Using cholera outbreak data from Bengal from 1891–1940, King et al. [9] estimated the basic reproductive number (R_0_, the average number of secondary cases produced by an index case of an infectious disease introduced into an immunologically naïve population [10]) to be approximately 1.6±0.3, while Hartley et al. [11] estimated an R_0_ ranging from 3 to 15 using average age of first infection and life expectancy data 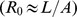 from four past epidemics between 1985 and 2001. Their analysis yielded an R_0_ of 18 for *V. cholerae* in a “hyperinfective” state. Similarly, the symptomatic attack rate of cholera is poorly estimated. Currently available studies suggest that the ratio of asymptomatic (or unrecognized) to symptomatic (or recognized) cases range from 3 to 100 [9].

One source of quantitative data on the dynamics of cholera epidemics that occurred without intervention, and which has not previously been explored, is *A Treatise on Epidemic Cholera* by the American psychiatrist Amariah Brigham [12]. Brigham published the book in 1832, the year cholera first appeared in North America, and although it is disheartening to read Brigham’s encyclopedic list of “remedies” for cholera (which included everything from bloodletting and blisters, to cautery, heated sand, and electricity), the book also includes quantitative appendices containing data that can be used to reconstruct epidemic curves, as well as data on case fatality for numerous cities in Europe and North America. We used Brigham’s data to parameterize simple mathematical models of cholera transmission; our objectives were (i) to derive R_0_ using the Richards generalized logistic growth model, (ii) to estimate projected ratios of recognized:unrecognized cases using Kermack and McKendrick’s formula for final epidemic size, and (iii) to examine the utility of historical data for the parameterization of models of cholera spread.

## Results

**Figure 1:**
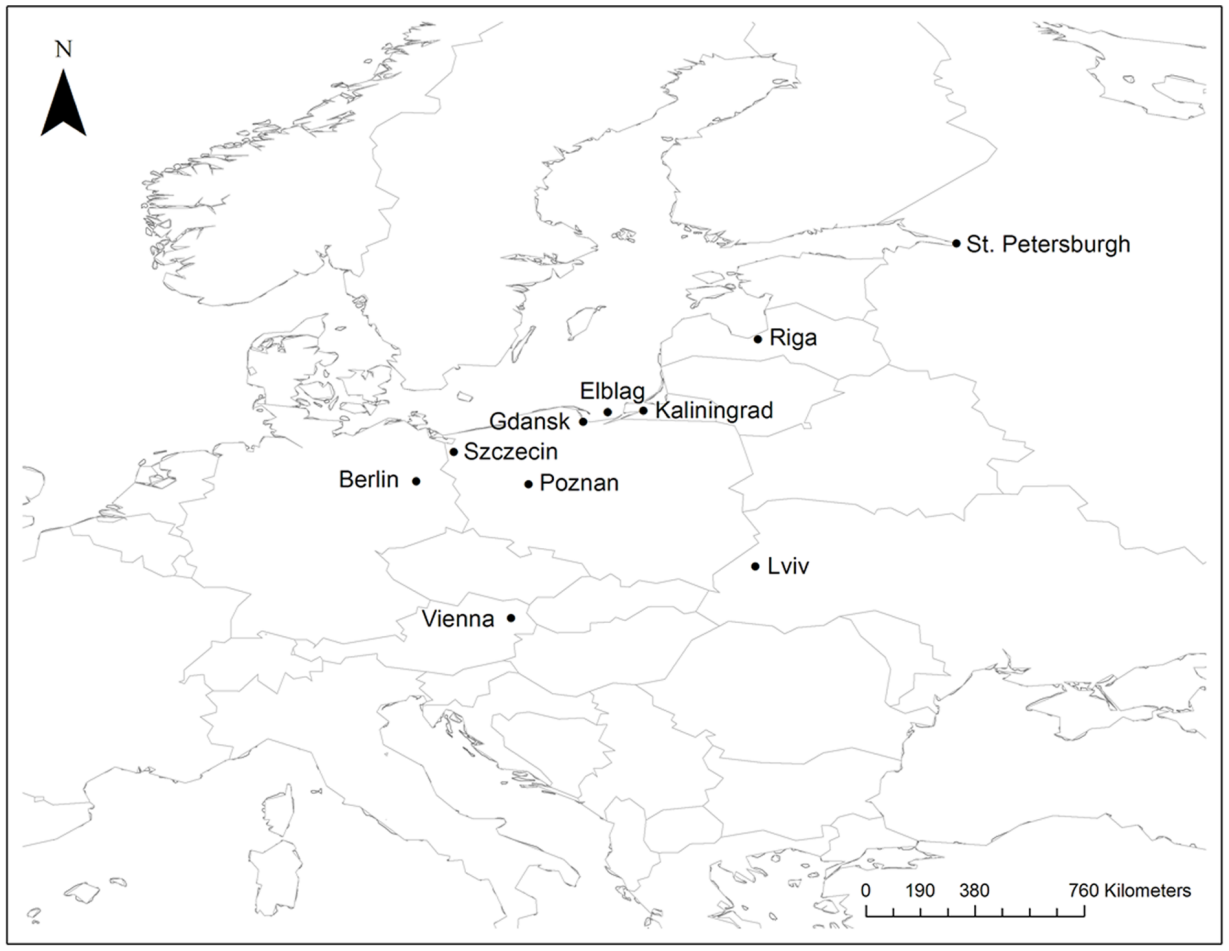
Map showing the locations of cities with time-series data on the 1832 cholera epidemic available in Amariah Brigham’s *A Treatise on Epidemic Cholera*. New York and Philadelphia are not shown.

Time-series data from a total of 12 cities were analysed. The ten European cities were all major port cities or centers for commerce at the time, with several cities being members of the Hanseatic League, a commercial alliance of trading cities surrounding the Baltic Sea formed in the 13^th^ century [13] (**Figure 1**). The North American cities of Philadelphia and New York were included. Population, death counts and case counts from Brigham’s *A Treatise on Epidemic Cholera* are shown in **Table 1**. Symptomatic attack rates and case fatality rates derived from Brigham’s data are also shown in **Table 1**. Weekly and daily case counts are presented in **Table 2** and **Table 3**.

**Table 1:** Cholera Cases and Deaths in Selected Cities, 1832.

City	Population	Deaths	Cases	Reported Attack Rate (%)	Reported Case Fatality (%)
Lemberg	45000	2622	5011	11.13	52.32
Riga	49000	1913	4897	9.99	39.06
Dantzig^a^	66367	1043	1432	2.15	72.83
Petersburgh	434000	4331	8803	2.02	49.19
Elbing	19225	245	378	1.96	64.81
Posen	30000	549	867	2.89	63.32
Konigsberg^a^	69560	1210	1996	2.86	60.62
Stettin^a^	21680	241	343	1.58	70.26
Berlin	230000	1384	2193	0.95	63.10
Vienna	290000	1895	3546	1.22	53.44
Philadelphia	161000	615	1710	1.06	35.96
New York	203000	2067	5319	2.62	38.86

a Cities with two-phase outbreaks.

**Table 2:** Weekly cholera case counts in various European cities, 1832.

Time (Week)	Lemberg	Riga	Dantzig	Petersburgh	Elbing	Posen	Konigsberg	Stettin	Berlin	Vienna
**1**	147	707	52	201	73	27	44	18	64	764
**2**	337	1331	87	1975	81	65	265	50	163	442
**3**	508	650	111	3492	36	124	346	59	336	391
**4**	774	635	153	1655	41	189	260	51	217	509
**5**	792	682	154	659	40	114	231	37	249	434
**6**	907	335	88	304	34	135	125	19	251	399
**7**	631	251	60	165	31	87	103	16	271	326
**8**	314	163	135	80	22	53	73	50	239	281
**11**	286	78	165	99	9	26	48	20	135	
**10**	105	65	167	84	6	33	63	23	141	
**11**	72		102	41	4	13	100		64	
**12**	50		60	30	1	1	111		63	
**13**	34		36	18			143			
**14**	23		18				84			
**15**	15		11							
**16**	12		22							
**17**	3		8							
**18**	1		3							

**Table 3:** Daily cholera case counts in Philadelphia and New York, 1832.

Time(Day)	Philadelphia	New York	Time (Day)	New York(Cont’d)
**1**	2	7	**24**	122
**2**	6	18	**25**	145
**3**	6	24	**26**	122
**4**	15	85	**27**	103
**5**	19	42	**28**	121
**6**	21	105	**29**	86
**7**	40	109	**30**	81
**8**	35	129	**31**	90
**9**	45	119	**32**	88
**10**	65	101	**33**	96
**11**	176	115	**34**	101
**12**	136	133	**35**	89
**13**	114	163	**36**	82
**14**	154	146	**37**	73
**15**	142	138	**38**	97
**16**	126	202	**39**	76
**17**	110	226	**40**	67
**18**	130	311	**41**	105
**19**	111	241	**42**	42
**20**	73	231	**43**	75
**21**	94	296	**44**	79
**22**	90	157	**45**	63
**23**		141	**46**	77

**Figure 2:**
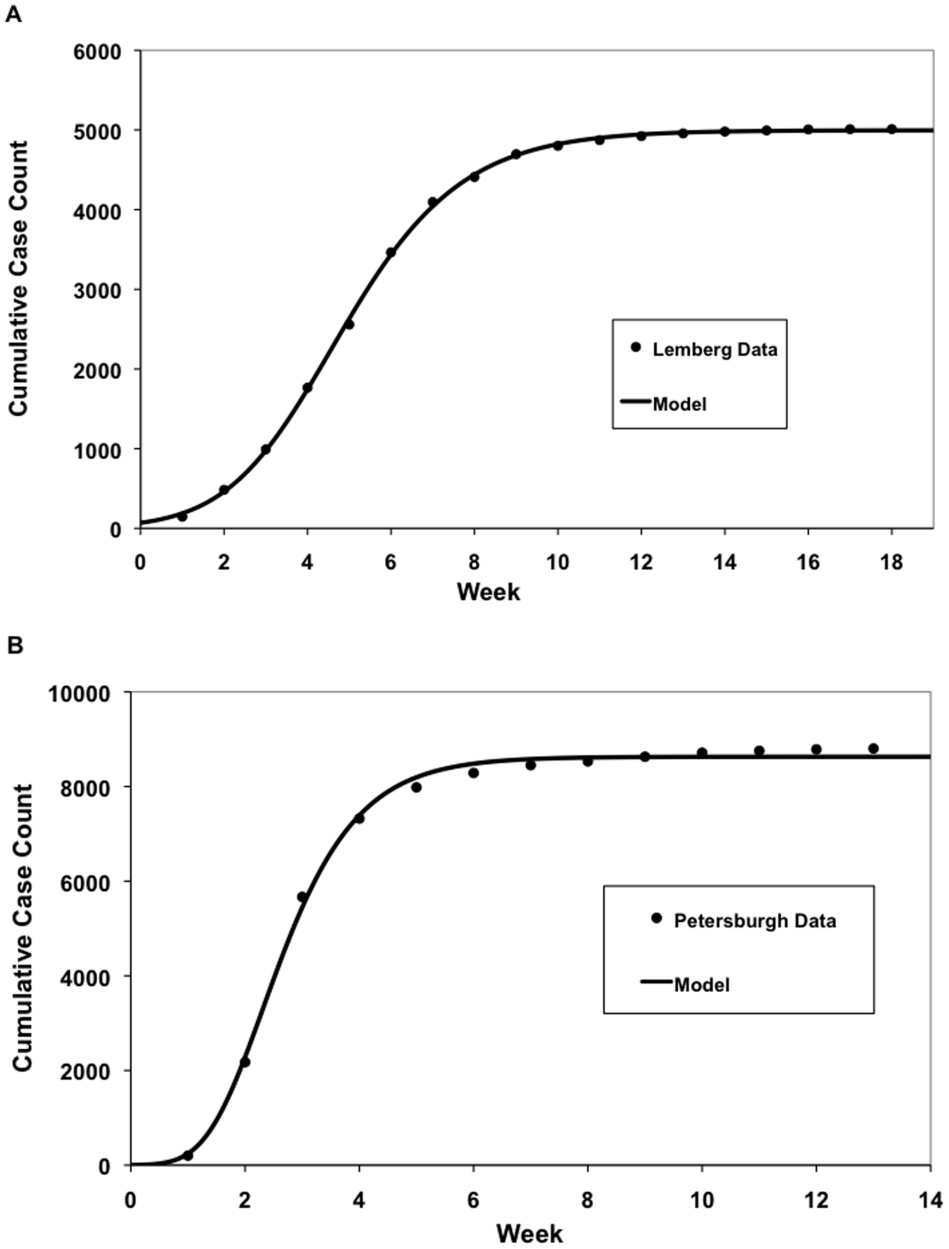
Selected curve fits of Richards curve to cumulative case count of cholera in the cities of (a) Lemberg (Lviv) and (b) Petersburgh (St. Petersburgh) reported in Brigham’s *A Treatise on Epidemic Cholera*.

**Figure 3:**
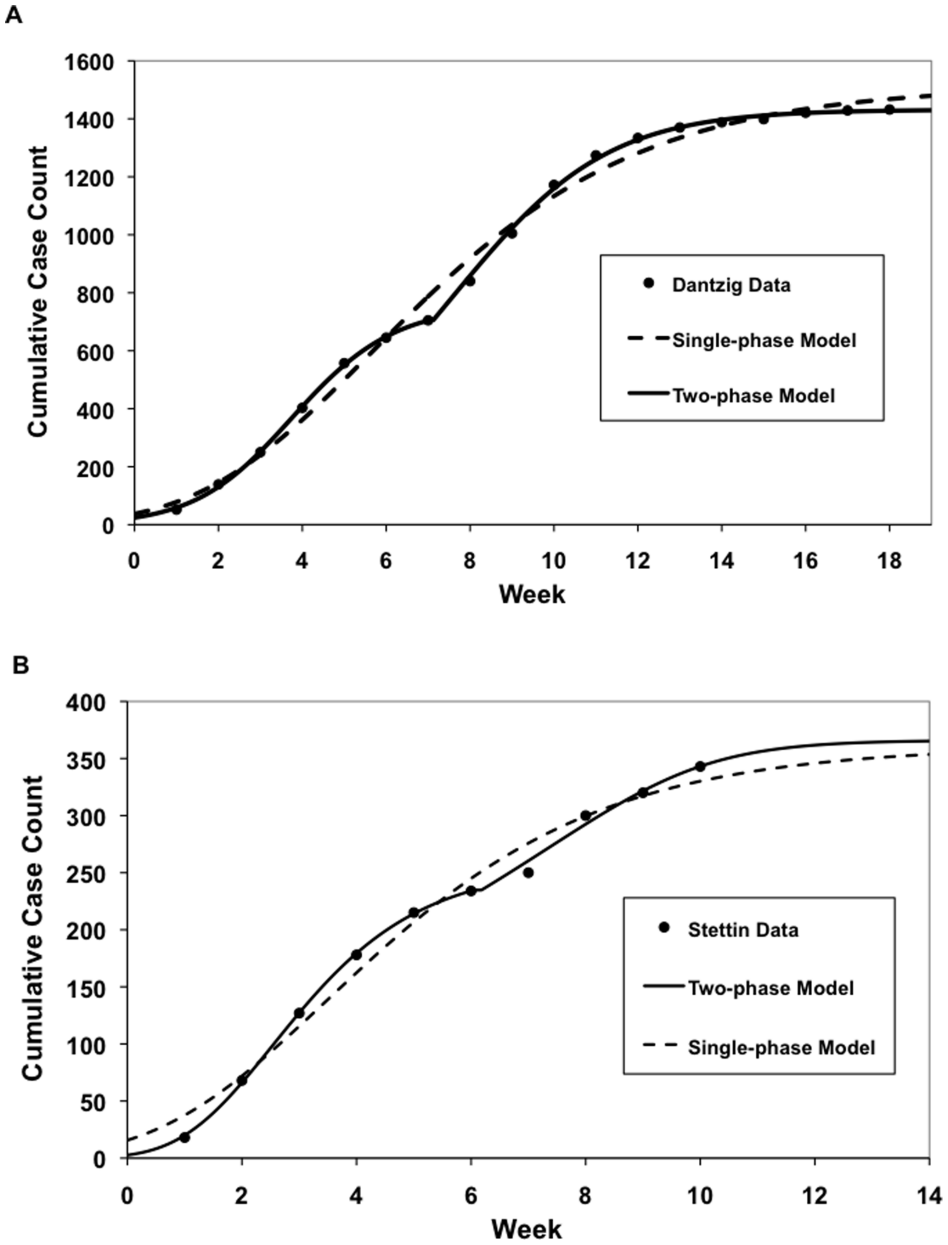
Selected curve fits of Richards curve to cumulative case count of cholera in the cities of (a) Dantzig (Gdansk) and (b) Stettin (Szczecin) reported in Brigham’s *A Treatise on Epidemic Cholera*. Results show poorer fits of the data with a single-wave outbreak model, whereas a two-wave outbreak model yielded better fits.

Overall, the Richards model fitted the cumulative weekly case count well (**Figure 2**). Because the model was first fitted under the assumption that the epidemic was single wave, it gave poor fits for Dantzig, Stettin and Königsberg, where cholera epidemics occurred in two waves, as evidenced by the observation that the cumulative incidence curve produced by the model did not fit the data well visually, and that two distinctive peaks could be seen in the epidemic curve produced by the data. Data from these cities were fitted again with a two-phase outbreak model [14], which yielded better fits (**Figure 3**).

Based on the single-phase models, the estimated value of R_0_ ranged from 1.93 (Lemberg) to 550.92 (Petersburg), with a median of 15.98 (11.85 if Petersburg is disregarded). Proportion of unrecognized cases calculated based on final size estimation and observed attack rates averaged at 0.96 (standard deviation, 0.04), which gives an average asymptomatic:symptomatic ratio of 48.29 (standard deviation, 29.72). The estimated values for intrinsic growth rate (r), R_0_, expected final size, percentage of unrecognized infections and ratios of unrecognized to recognized cases from each city are presented in **Table 4**.

**Table 4:** Model-Estimated R_0_, Expected Final Size of Epidemic, and Percentage of Asymptomatic or Unrecognized Cases.

City	r	R_0_	Expected Final Size (Proportion of population)	Percent Asymptomaticor Unrecognized	Asymptomatic/Unrecognizedto Symptomatic Ratio
Lemberg	1.152	1.93	0.77	85.53	5.915
Riga	5.363	21.4	0.99	89.90	8.906
Dantzig^a^	0.5592	1.38	0.48	95.50	21.25
Phase 1	0.9638	1.73			
Phase 2	0.3679	1.23			
Petersburgh	11.04	551	0.99	97.95	47.81
Elbing	4.921	16.6	0.99	98.01	49.35
Posen	5.265	20.2	0.99	97.08	33.26
Konigsberg^a^	4.775	15.3	0.99	97.10	33.50
Phase 1	6.190	34.4			
Phase 2	0.07147	1.04			
Stettin^a^	3.722	8.39	0.99	98.40	61.57
Phase 1	6.940	52.8			
Phase 2	0.5979	1.41			
Berlin	3.512	7.44	0.99	99.03	102.8
Vienna	1.617	2.52	0.90	98.64	71.79
Philadelphiab,c	0.9239	40.3	0.99	98.92	92.21
New York^c^	1.253	150	0.99	97.35	36.78

a Cities with two-phase outbreaks: Expected final size, percent asymptomatic or unrecognized cases and asymptomatic/unrecognized to symptomatic ratio were calculated using the R_0_ estimated from fitting the single-phase model to the epidemic curve.

b Philadelphia data is from second wave of outbreak.

c Cases were counted daily in these cities.

It should be noted that for cities that had a two-phase epidemic, the R_0_ estimation from the second phase of the epidemic is somewhat meaningless, since the initial condition assumption of I(0) = 0 does not hold at the beginning of the second phase. The expected final size and percentage of unrecognized infections were therefore estimated based on the R_0_ produced from fitting a single-phase model to the epidemic curve.

As expected, sensitivity analysis showed that increasing the infectious period (T) leads to an exponential increase in the estimated R_0_ values [as shown in Supplementary Figure S1]: the larger the modeled intrinsic growth rate (e.g. Riga), the greater the variation, while estimated R_0_ values remained relatively stable when the intrinsic growth rate is smaller (e.g. Philadelphia).

## Discussion

We evaluated the dynamics in European and American centers during the second cholera pandemic using mathematical modeling techniques that can be applied to cumulative incidence data. R_0_ estimates generated through modeling were greatly heterogeneous, though such heterogeneity is consistent with variability in cholera dynamics described by contemporary investigators. Possible sources of such heterogeneity could include differential data quality, or true differences in the nature of cholera spread within the cities. Higher R_0_ values may point to gross contamination of water sources, while lower R_0_ values might indicate predominantly person-to-person transmissions.

We also evaluated gaps between reported cumulative cases reported in each jurisdiction, and the projected final epidemic sizes expected based on reproductive numbers, in order to develop estimates of the ratio of unrecognized to recognized cholera cases. As this calculation was a function of R_0_ estimates it is perhaps unsurprising that we found these ratios to be heterogeneous as well. While it is possible that misclassification and underreporting of cholera could have been major sources of error in the data it should be noted that such misclassification would not have affected our estimates of R_0_: the Richards model evaluates rate (and hence R_0_) of growth in a manner that is independent of case counts reported. Distortion of R_0_ values could have occurred if case ascertainment changed over time in a given center; however, to explain the diversity of R_0_ values such changes would have had to have occurred in different ways in each city we studied, and consequently true variability in epidemic growth remains plausible.

Variation in case measurement across cities would have been more likely to explain heterogeneity in apparent ratios of unrecognized to recognized case counts. Cholera was a highly stigmatized in the 1830s, and was seen as a disease that struck the poor and immoral as a punishment from God [15]. Cases of cholera could have been underreported among communities that were supposedly immune to the disease, such as ostensibly temperate members of the establishment. Furthermore, the background level of diarrhoea and dysentery in communities is likely to have been high at the time [16], [17], [18]. Bacteriological diagnosis of cholera awaited the work of Robert Koch in the 1880s [19], hence the ability of clinicians to distinguish between “Asiatic cholera”, so-called *cholera morbus* (basically endemic watery diarrhea [1]), and other forms of gastrointestinal disease which shares the symptoms of Asiatic cholera was also limited. Since most cases of cholera only result in mild or asymptomatic cases, many of them were likely to have been misclassified as well.

Even though there is much uncertainty about the reliability of Brigham’s data, the R_0_ values produced in this study are for the most part remarkably similar to R_0_ estimates from studies using more detailed transmission models and greater number of parameters. King et al. estimated an R_0_ of 1.6±0.3 for endemic cholera in the Bengal region when taking into consideration environment-human transmissions and human-human transmissions [9]. Postulating the existence of a “hyperinfective” stage for cholera, Hartley et al. estimated an R_0_ of 18 for the disease in this stage [11]. Our estimated unrecognized to recognized case ratios are also consistent with those generated using contemporary data (which are also highly variable) [9].

Our model represents, to our knowledge, the first application of the Richards growth model to evaluate the dynamics of historical epidemics. The Richards model, while long known in ecology and population biology, has had relatively recent application to infectious diseases, but has a number of attractive properties, including its use of a single equation, its ready parameterization when (as in this case) only epidemic curve data are available, and the easy interpretation of model parameters (such as growth rate, carrying capacity/final size, and “turning point”) in terms that have direct relevance to disease control practice. The model has been applied to contemporary infectious disease outbreaks and epidemics, including those caused by influenza, Severe Acute Respiratory Syndrome (SARS), and dengue [14], [20], [21]. Model generated case counts produced with the model for cities under study fit remarkably well with case counts reported by Brigham.

Our complementary use of the Richards growth model and the Kermack-McKendrick final size formula emerges as a potentially useful index of the proportion of cases that are unrecognized or truly asymptomatic in situations where effective interventions are not put in place for epidemic control. The lack of understanding of the role of contagion and contaminated water in the spread of cholera in 1832 makes the situation under study one in which this approach is likely to be valid, and this approach may be particularly attractive for the study of historical epidemics where control measures were ineffective. In contemporary epidemics with control, comparison of final epidemic size with expected final size projections based on the Kermack-McKendrick formula would have a different (but also useful) interpretation, and would be a function of both unrecognized/asymptomatic case rates and the effectiveness of control measures.

We propose that the ease of use of the Richards model, and the face validity of the disease dynamic properties generated here for cholera, suggest that this tool may find application in the study of historical epidemics. While subject to limitations associated with measurement and the absence of microbiological diagnosis, historical epidemic data are potentially extremely attractive for study for several reasons: as with early cholera epidemics, the absence of effective control measures means that observations relate to the transmission dynamics of the pathogen itself. Furthermore, advances in disease control through sanitation and vaccination have made many communicable diseases of public health importance uncommon, making the study of epidemic dynamics with contemporary data difficult. For example, Earn has previously demonstrated that regular epidemic cycles become chaotic once vaccination is introduced [22]. The study of historical epidemics can also provide important insights into the impact of economic development on health. With advances in public health and improvement in living conditions, cholera has practically disappeared from Europe and North America, save for a few imported cases each year [3]. As our data demonstrate, early 19^th^ century cholera epidemics in Europe and North America had properties similar to those seen in low income countries today [23], [24], [25], highlighting the fact that it is not intrinsic characteristics of populations, but rather economic conditions, that determine vulnerability to disease.

Our study has limitations, most of which relate to the temporally distant nature of the epidemics under study, and the lack of clarity in Brigham’s document with respect to the conditions under which data were collected. Nonetheless, it shows an innovative use of the Richards model and the Kermack-McKendrick final size formula in conjunction with historical data that help illuminate the dynamics of cholera transmission in the 2^nd^ pandemic in 19^th^ century Europe and North America. Our study also highlights the potential of the Richards model to be an effective tool for modeling infectious diseases.

## Methods

### Data Sources

Dr. Amariah Brigham (1798–1849) was an American clinical physician known mainly for his work in psychiatry. When cholera spread across North America in 1832, he was residing in Hartford, Connecticut [26]. He took it upon himself to compile information on the origin and progress of the epidemic from reports, treatises, lectures and essays, published as *A Treatise on Epidemic Cholera*, in order to “furnish a correct history of the disease, together with all the most important practical information that has been published respecting its nature, cases and methods of treatment” [12].

In the appendix of the volume, Brigham had included time-series data on cholera in various European and North American cities. Weekly case counts were made available for Lemberg (now Lviv, Ukraine), Riga, Dantzig (Gdańsk, Poland), Petersburgh (Saint Petersburg, Russia), Elbing (Elbląg, Poland), Posen (Poznań, Poland), Königsberg (Kaliningrad, Russia), Stettin (Szczecin, Poland), Berlin and Vienna, and daily case counts were provided for Quebec, Montréal, New York and Philadelphia. Quebec and Montreal were excluded from the present analysis due to aggregation of case counts early in the epidemic (for Montreal) and the reporting of hospitalized cases only (for Quebec).

### Epidemic Growth Model

The Richards growth model is a generalized logistic growth describing biological growth [27]. It has been used in infectious disease epidemiology as a method to estimate R_0_ and forecast epidemics with SARS and dengue [20], [21]. The intrinsic growth rate of an epidemic can be estimated by fitting the cumulative time-series case data from an epidemic curve to the Richards model: 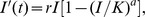 where *r* is the intrinsic growth rate of the infected population, *I*(*t*) is the cumulative case count at time *t*, *K* is the final total case number of the outbreak, and *a* measures the deviation of the curve from the standard logistic curve [21]. The advantage of the Richards model is that it involves relatively few parameters and only requires an epidemic curve for analysis, which proves to be convenient when data availability is limited.

The intrinsic growth rate derived from Richards model can be used to calculate the basic reproductive number (R_0_) of a disease [14], [28]. In general, when R_0_<1, the infection is expected to eventually disappear from the population, and when R_0_>1, then one would expect an epidemic of the infection to occur. Using the estimated value of *r* from the Richards model, R_0_ can be estimated with 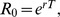 where *T* is the duration of infectiousness [14]. For cholera, the infectious period is estimated to be 3–6 days [29], and is assumed to be 4 days in our analysis. A sensitivity analysis was performed to explore the effect of varying the infectious period from 3 to 6 days on the values of R_0._ Best-fit model parameters were estimated empirically via least squares minimization for model projections and reported case counts.

The Richards model is generally suited for single-phase epidemics [28], [30], but can be adapted to model multi-wave epidemics as well [14]. In a two-phase outbreak model, the Richards model is fitted to the cumulative incidence curve from the first wave of the epidemic, and then again to that from the second wave. We applied the two-phase outbreak model in cases where the epidemic curve showed two distinctive peaks in the course of the epidemic. In our study, the turning point between the first and second wave of cholera outbreak was determined by examining the incidence epidemic curve. It was identified as the point at which there was a rebound in case counts after a decline from the first peak to reach a second peak. Two sets of best-fit model parameters were obtained, one from each phase of the epidemic.

R_0_ estimates derived from Richards models were used to project final epidemic sizes using the final epidemic size formula first published by Kermack and McKendrick [31]. Under the SIR model proposed by Kermack and McKendrick, an epidemic ceases when a certain proportion of the susceptible population has been infected (not necessarily the entire population), even without intervention (as was, effectively, the case during the 2^nd^ cholera pandemic). This proportion (Z) is dependent on the R_0_ of a disease, and is defined by the equality 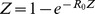 [32]. Using the expected final sizes and the observed attack rates, the proportion of unrecognized (asymptomatic or undiagnosed) cases can be calculated, and thereby the ratio of unrecognized cases to symptomatic, recognized cases of cholera can be determined. Few assumptions are made by the Richards model: 1) the rate of increase in cumulative case is proportional to present number of cases, 2) case incidence grows exponentially without intervention and 3) effective interventions would decrease incidence growth [28]. Cholera satisfies all three assumptions.

In the original publication of Kermack and McKendrick’s final size formula, two major assumptions were made: that the infectious periods of the disease are exponentially distributed, and that the host population is homogenously mixed. Ma and Earn showed that the formula still holds “(i) regardless of the number of distinct infectious stages, (ii) if the mean contact rate is itself arbitrarily distributed and (iii) for a large class of spatially heterogeneous contact structures” [32]. For two-wave epidemics, this approach is problematic since I(0) is not equal to zero for the second wave. Consequently, we approximated expected final size for two-wave epidemics based on the best-fit single epidemic curve generated using the Richards approach.

## Supporting Information

Figure S1 Sensitivity analysis on the impact of estimated cholera generation time on cholera R_0_. City names in legend are ordered from highest R_0_ (Riga) to lowest (Philadelphia). A 4-day generation time was used in the base case. Cities with an asterisk next to name had 2-wave cholera epidemics; best-fit single wave R_0_ estimates are presented here. It can be seen that as expected the absolute impact of uncertainty in generation time is greatest for high-R_0_ cities.
(TIF)
