# Supplementary figures and images for: Historical Epidemiology of the Second Cholera Pandemic: Relevance to Present Day Disease Dynamics
Source: PLoS One. 2013 Aug 22;8(8):e72498. doi: 10.1371/journal.pone.0072498 (PMC3749991; doi:10.1371/journal.pone.0072498)

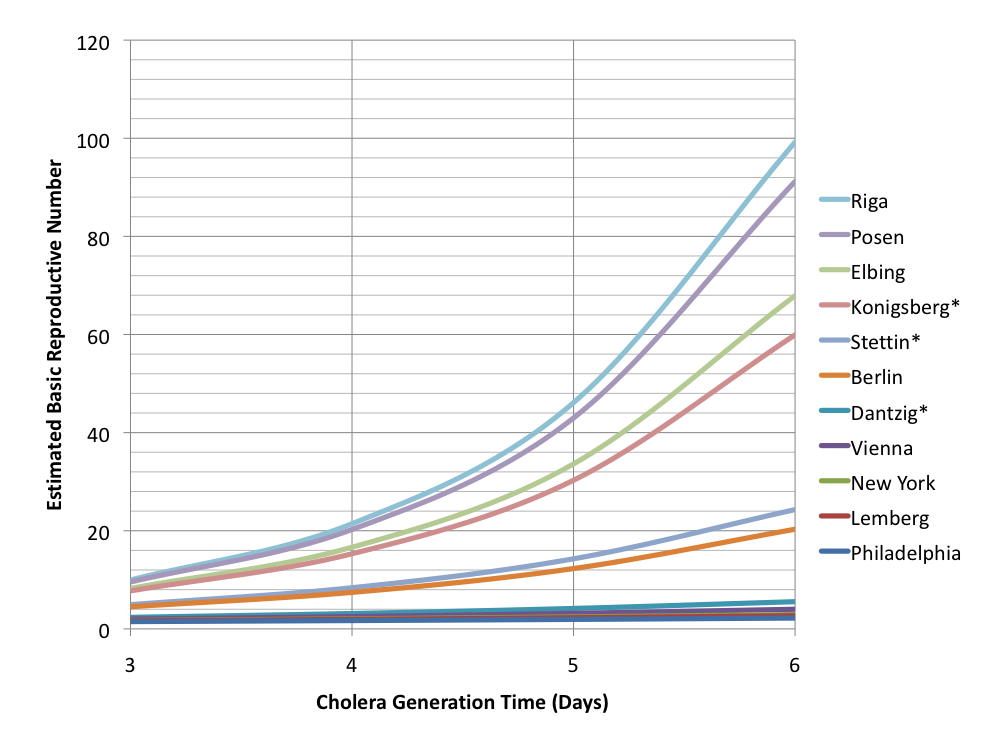

Supplement: Figure S1 — Sensitivity analysis on the impact of estimated cholera generation time on cholera R0. City names in legend are ordered from highest R0 (Riga) to lowest (Philadelphia). A 4-day generation time was used in the base case. Cities with an asterisk next to name had 2-wave cholera epidemics; best-fit single wave R0 estimates are presented here. It can be seen that as expected the absolute impact of uncertainty in generation time is greatest for high-R0 cities. (TIF) [file pone.0072498.s001.tif]
